# The past, present and future of HIV, AIDS and resource allocation

**DOI:** 10.1186/1471-2458-9-S1-S4

**Published:** 2009-11-18

**Authors:** Steven Forsythe, John Stover, Lori Bollinger

**Affiliations:** 1 Futures Institute, 41-A New London Tpke, Glastonbury, CT 06033, USA

## Abstract

**Background:**

How should HIV and AIDS resources be allocated to achieve the greatest possible impact? This paper begins with a theoretical discussion of this issue, describing the key elements of an "evidence-based allocation strategy". While it is noted that the quality of epidemiological and economic data remains inadequate to define such an optimal strategy, there do exist tools and research which can lead countries in a way that they can make allocation decisions. Furthermore, there are clear indications that most countries are not allocating their HIV and AIDS resources in a way which is likely to achieve the greatest possible impact. For example, it is noted that neighboring countries, even when they have a similar prevalence of HIV, nonetheless often allocate their resources in radically different ways.

These differing allocation patterns appear to be attributable to a number of different issues, including a lack of data, contradictory results in existing data, a need for overemphasizing a multisectoral response, a lack of political will, a general inefficiency in the use of resources when they do get allocated, poor planning and a lack of control over the way resources get allocated.

**Methods:**

There are a number of tools currently available which can improve the resource-allocation process. Tools such as the Resource Needs Model (RNM) can provide policymakers with a clearer idea of resource requirements, whereas other tools such as Goals and the Allocation by Cost-Effectiveness (ABCE) models can provide countries with a clearer vision of how they might reallocate funds.

**Results:**

Examples from nine different countries provide information about how policymakers are trying to make their resource-allocation strategies more "evidence based". By identifying the challenges and successes of these nine countries in making more informed allocation decisions, it is hoped that future resource-allocation decisions for all countries can be improved.

**Conclusion:**

We discuss the future of resource allocation, noting the types of additional data which will be required and the improvements in existing tools which could be made.

## Background

In 2001, demographers, epidemiologists and economists, under the direction of the World Health Organization and UNAIDS, were asked to estimate the resources required to achieve the newly developed UNGASS targets [1]. The $9.2 billion projection represented an optimistic estimate of the level of resources which could be generated by 2005, considering that the global level of resources for HIV and AIDS were only $1.6 billion in 2001.

**Figure 1:**
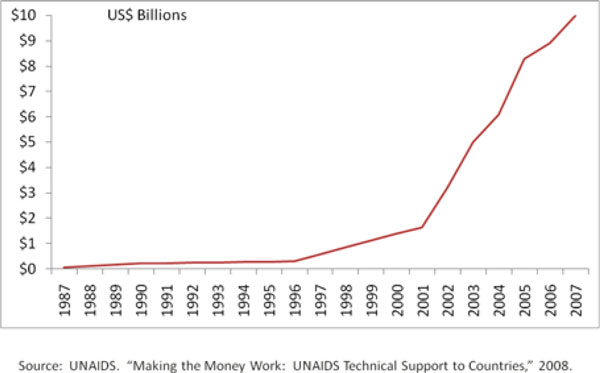
**Global HIV and AIDS Spending (1987-2007)**.

One of the great successes in raising funds for the response to the HIV and AIDS pandemic has been the rapid increase in resources for the global response, as shown in Figure 1. From the UN General Assembly Special Session on HIV and AIDS in 2001 to the most recent estimates of global spending in 2007, the level of funding has grown more than 6 fold to $10 billion [2]. The actual level of spending in 2005 reached $8.3 billion [2], or 90% of the resources determined to be required in the original 2001 resource needs estimates. Estimates of global resource needs have been reassessed on a regular basis. The most recent assessment projects needs in 2015 of US$22-54 billion, under three different assumptions about the pace of scale-up [2].

While no single factor can explain why the growth in spending has occurred so rapidly, it can be partially explained by a number of critical events, including:

• The launch of UNAIDS in 1996;

• The introduction of the World Bank MAP program, and the Bill and Melinda Gates Foundation in 2000;

• The UN General Assembly Special Session on HIV and AIDS (UNGASS) in 2001;

• The start of the Global Fund in 2002;

• The launch of PEPFAR in 2003;

• The G8 Summit in 2005 and the corresponding commitment to universal access.

While the actual resources spent in 2005 came relatively close to the target of $9.2 billion, it is important to remember that the real goal was to scale up the delivery of services and to change behaviors, not to generate new resources.

A review of the actual achievements relative to the expected achievements is not as impressive as the scale up of resources. The 2006 Report on the Global AIDS Epidemic concluded that of 6 targets evaluated, only one was achieved: the generation of between US$7-$10 billion in funding by 2005 [3]. For example, one target was to have 90% of young people be able to identify ways of preventing HIV transmission and to reject major misconceptions about HIV transmission. On average, only 33% of males and 20% of females met this requirement in UNGASS reporting countries; none of the countries were able to achieve the targeted level of 90% coverage. Another target was that there would be 80% coverage of PMTCT programs, a target which was not achieved by any of the reporting countries in 2005.

The original resource requirement estimates were based on an assumption that 6 billion condoms would be distributed annually; by 2005, 5 billion condoms were actually distributed (83% of the original target) [4]. Reaching vulnerable populations proved to be more difficult, however; only 61% of the estimated number of sex workers targeted were actually reached, while only 37% of those injecting drug users (IDU) who were assumed to be reached with the resources were actually reached. Difficulties were also encountered in providing care: only 31% of those targeted to receive antiretroviral therapy (ART) were reached and only 28% of the PMTCT target was actually achieved. The one target that was achieved was the number of people receiving VCT services, which succeeded in reaching 185% of the original estimate. In other words, despite generating 90% of the required resources, most of the targets used to calculate resource requirements were not achieved by 2005 [4,5].

Thus, while the global response did succeed in rapidly generating new resources, it did not succeed in fully generating the services and the behavior change which was anticipated. This suggests that either the original projections were overly optimistic about what could be achieved with the resources, or the world spent its HIV and AIDS resources in a much more ineffective (or inefficient) way than what was originally assumed.

Given the current financial crisis and the potential for donors to redirect their attention and resources to other priorities, it is important to assess how current resources have been spent and ask how resources might be allocated to assure the achievement of targets in the future. How can resources be spent to reach those groups most at risk? Will the allocation of resources be consistent with what is known about the effectiveness of interventions? Can the positive response of the past decade be sustained even if the rapid growth in resources does not continue?

## Objective

This paper was developed with three objectives in mind. The first objective is to identify some of the challenges and inconsistencies in the ways current HIV and AIDS resources are allocated at the national level. This section of the report will define an "evidence-based allocation strategy" and assess if countries are making allocation decisions based on such evidence.

We will also look at how countries are currently spending their HIV and AIDS resources, with a particular focus on whether these current allocation patterns appear to be more driven by politics and wishful thinking, or if they are more driven by evidence and careful planning. This section will also discuss why some countries may not be following an evidence-based allocation strategy.

The second objective is to review some of the national-level experiences in trying to improve the resource-allocation process. We will address this objective by identifying and explaining the experience of groups using evidence-based tools, such as the Resource Needs Model (RNM) [6] and the Goals Model [7].

The third objective is to assess how resource allocation in the future might be improved through encouraging countries to pursue an "evidence-based allocation strategy". This section also discusses how current and future tools might be improved to address the growing need for better resource allocation.

## What is an evidence-based allocation strategy?

The simplest definition of an "evidence-based allocation strategy" is one in which resources are spent in a way that is, based on the best currently available evidence, likely to achieve the greatest possible result. In the case of HIV and AIDS, results are generally defined in terms of preventing new infections, providing care and treatment, and mitigating impact.

### How should HIV and AIDS resources be spent?

An ideal approach for assessing how policymakers should optimally allocate resources would involve comparing a country's actual spending patterns on HIV and AIDS to some known optimal allocation pattern. Countries which differed significantly from the optimal allocation pattern would be considered to need to reallocate their funds. Countries closest to the optimal allocation would be the ones which would not need to reallocate (and presumably would be provided with more money by donors in recognition of allocating their resources effectively).

For example, perhaps Country A is currently spending only 2% of its HIV and AIDS prevention resources on reaching injecting drug users (IDU). The country's 5 year HIV and AIDS strategy indicates that IDU are not a priority and will continue to receive only a small proportion of all prevention resources. However, the optimal allocation pattern suggests that IDU are an important driver of the epidemic and Country A should be spending more than 20% of its resources on this population. In this case, policymakers might be encouraged to rethink their plans and increase spending on this critical subpopulation. If the misallocation was not corrected, the country might receive less money from international donors in the future, since the country is not allocating its resources effectively

*"Many governments have not acted in a fully responsive manner to protect their populations from HIV infection or death from HIV disease. Many countries, rich and poor, still fail to devote adequate resources to address their national epidemics, choose not to implement evidence-based programming, or ignore the needs of marginalized groups affected by HIV" *[8].

Alternatively, Country B has pursued an "evidence-based allocation strategy", carefully assessing its epidemic, the cost-effectiveness of its interventions, synergies between interventions, the policy environment, etc. Using this evidence, Country B has established clear and logical priorities and has allocated its resources accordingly. In this case, donors may choose to assign more funds to Country B, because that country is more likely to have an impact on its epidemic.

Unfortunately at this time, calculating this optimal allocation pattern is not entirely feasible, in part because there is no known optimal allocation of HIV and AIDS resources [9]. With the availability of better prospective information on spending patterns and HIV prevalence, it may become possible in the near future to design optimal allocation strategies, based on what has worked to reduce prevalence/incidence, increase treatment, and minimize the impact of HIV and AIDS. Even if an optimal allocation pattern did exist, it would need to be significantly modified for each individual country to reflect differences in HIV prevalence and/or incidence levels, modes of HIV transmission and unit costs.

Even countries with similar epidemics, similar modes of transmission and similar unit costs might choose to allocate their resources in different ways due to cultural and political differences (e.g., some countries might place a higher weight on equity, for example, while other countries might emphasize cost-effectiveness). This is not to suggest that making decisions based on the political reality which exists in a country is wrong. In fact, in countries where behaviors such as homosexuality are illegal, it is particularly problematic to design programs which will successfully reach MSM. In this case, it might make more sense to first spend the limited resources to address the legal environment and then to scale up resources for MSM.

However, even considering the current lack of data, one would still expect some consistency in the way resources are allocated, based on the epidemiological and economic data available. If resource-allocation decisions are actually being informed by an evidence-based allocation strategy rather than being made randomly or based on purely political factors, then one might expect that neighboring countries, with a similar prevalence of HIV, would allocate their resources in somewhat similar patterns. Is this the case?

### How are resources currently being spent?

With the advent of National AIDS Spending Assessments (NASA), much more is currently known about the ways in which HIV and AIDS resources are being spent [10]. The 2008 UNAIDS Global Report on the epidemic provides an informative, albeit imperfect, summary of HIV and AIDS spending from country to country [11]. This provides a unique opportunity to analyze for the first time how HIV and AIDS resources are currently being spent. Is money being spent on those interventions which will achieve the greatest possible impact, or are the resources being randomly allocated to various interventions, based on the hope that everything will have some impact?

**Figure 2:**
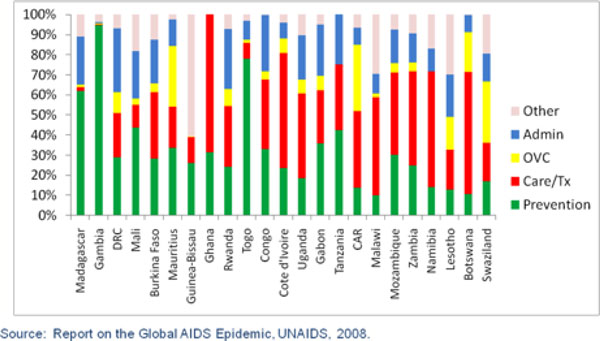
**Allocation of HIV/AIDS Resources in sub-Saharan Africa**.

**Figure 3:**
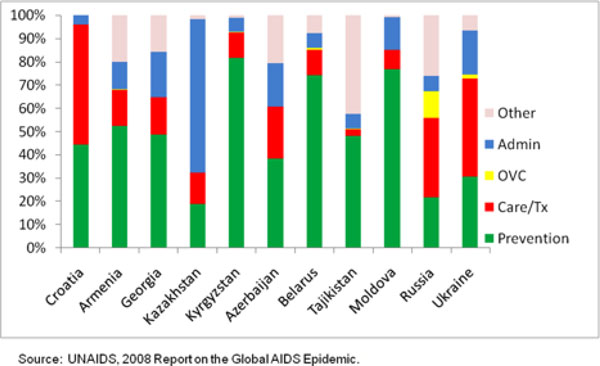
**Distribution of HIV Expenditures in Eastern Europe/Central Asia**.

**Figure 4:**
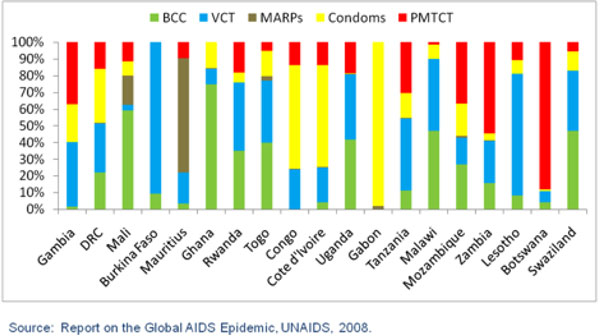
**Allocation of Prevention Resources in sub-Saharan Africa**.

Figure 2 shows the reported expenditure of HIV and AIDS funds in sub-Saharan Africa. The list of countries is ordered from the lowest prevalence country (Madagascar, with an adult prevalence of 0.1%) to the highest prevalence country (Swaziland, with an adult prevalence of 26.1%). Note that Figures 2, 3 and 4 were developed to show the proportions of all resources spent on different interventions. The graphs don't show the absolute amount of spending, which may influence how countries allocate their funds.

As the figure shows, lower prevalence countries allocate a higher proportion of their resources to prevention, while higher prevalence countries spend a larger percentage on care and treatment. For example, the eight countries with prevalence below 2% spend 38% of their resources on prevention, and only 23% on care and treatment. Meanwhile, the five countries with a prevalence of over 15% spend 17% of their resources on prevention and 48% on care and treatment. This allocation is consistent with what one might expect; low-prevalence countries have a lower burden of treatment and therefore allocate a greater share of their resources to prevention. High-prevalence countries should be spending heavily on prevention but will have much greater need for treatment. "Other HIV expenditures" in Figures 2 and 3 include human resources, social protection and social services, an enabling environment and research.

However, it is also worth noting that there appear to be some significant outliers. For example, the DRC reports spending 32% of its resources on administration, almost twice as much as the average for all 23 countries. Ghana, with a prevalence of only 1.9%, reports spending the highest proportion of any country on care and treatment (69% of all resources). Even when comparing two high-prevalence countries, such as Botswana (prevalence of 24%) and Swaziland (prevalence of 26%), the resource-allocation patterns differ significantly. Botswana is currently spending the largest proportion of its resources on care and treatment, while Swaziland is spending most of its resources on orphans and vulnerable children. As a result, Botswana has 76% coverage with ART in 2006, while Swaziland had only 35% ART coverage [11].

The allocation of resources appears to be even more random in Eastern Europe and Central Asia, as shown in Figure 3. The lowest prevalence country, Croatia, spends the largest proportion of its funds on care and treatment, which is contrary to what would be expected. Moving from the lowest prevalence countries on the left to the highest prevalence countries in the region on the right, there appears to be little consistency or pattern in the way resources are spent. Even neighboring countries with a similar prevalence of HIV, such as Kazakhstan and Kyrgyzstan, spend their HIV and AIDS resources in radically different ways. Kazakhstan spends most of its resources on program support, whereas Kyrgyzstan spends most of its resources on prevention.

The analysis becomes even more revealing when specifically evaluating the allocation of resources on prevention programs. In Figure 4, sub-Saharan countries are again arranged from the lowest-prevalence countries on the left to the highest prevalence countries on the right. In this case, the allocation of prevention resources appears to occur in a random pattern. Again looking at the two highest prevalence countries, Botswana and Swaziland, the approach to allocating prevention resources appears to be markedly different. In the case of Botswana, most prevention resources are spent on PMTCT, whereas Swaziland appears to spend a small proportion of its resources on this intervention. On the other hand, Swaziland appears to spend most of its resources on BCC and VCT, two interventions which are allocated a much smaller proportion of all resources in Botswana.

There are also numerous examples cited of misallocations of funds in Asia, as reported in Elizabeth Pisani's book "The Wisdom of Whores" [12]. For example, in China, 90% of HIV transmission is attributable to MSM or IDU, yet 54% of all donor prevention money is allocated to the "general population".

National policymakers are not the only stakeholders who appear to misallocate funds. In Accra, Ghana, for example, it has been estimated that 76% of all new HIV infections occur between sex workers and their partners, while the remaining 24% of all new infections occur within the general population [13]. However, the World Bank MAP program in Ghana estimates that it spends only 0.8% of its resources on sex worker interventions and spends 99.2% of HIV and AIDS resources on the general population [14]. These data suggest that donors, at least in the case of Ghana, also apparently failed to allocate their own resources based on evidence regarding the main drivers of the epidemic.

One caveat is worth noting in regards to the appropriate allocation of funds. If the cost to reach the general population and to change their behavior is much higher than the cost of reaching and changing the behavior of sex workers, it may be quite logical to allocate more funds to the general population than sex workers. In other words, an appropriate allocation of funds takes into consideration not only where new HIV infections are occurring, but also considers unit costs of reaching each population.

Reviewing the ways in which countries allocate their resources, it becomes difficult to demonstrate that countries are actually pursuing an "evidence-based allocation strategy". Neighboring countries with similar HIV prevalence levels spend resources in radically different ways. Spending patterns appear to be only nominally related to the severity of the epidemic in sub-Saharan Africa, and are totally unrelated to prevalence in the rest of the world. Prevention resources appear to be allocated randomly, with no evidence showing that countries allocate their spending based on evidence about the source of new HIV infections.

### Why don't countries allocate resources based on evidence?

If countries are not pursuing an "evidence based allocation strategy", the next question is to ask why. A number of possible explanations are listed below.

#### Lack of data

One possible explanation for poor resource allocation is that countries don't have access to the information required to make rational decisions regarding the allocation of HIV and AIDS funds. This is a very plausible explanation, given that most countries don't have access to data about the cost-effectiveness of different interventions. While there are data which suggest that some interventions may be more cost-effective than others [15-17], these data are often inadequate for the purpose of policymakers. For example, some studies were conducted in one region, while policymakers in a second region are hesitant to accept these data as being applicable.

However, the lack of data cannot fully explain the path some countries take in allocating resources. Even when data are available and reliable, it has been our experience that they are only used to a limited extent when make planning decisions.

#### Contradictory results

When effectiveness and/or cost-effectiveness data do become available, there are often contradictory messages derived from these results. For example, early economic analysis suggested that PMTCT interventions would not be affordable in South Africa [18]. Subsequent analyses concluded that not only would PMTCT be affordable, but it would also be cost-effective and potentially cost-savings in South Africa [19-21].

Retrospective data from Uganda about the country's success in reducing HIV prevalence rate also has produced contradictory results. In some cases, it has been suggested that prevalence reductions were attributable to behavior change which occurred as a result of a rapidly scaled-up VCT program [22]. However, other studies have suggested that prevalence reductions occurred as a result of the country's "zero grazing" campaign [23]. Still other studies suggest that the initial behavior change didn't occur because of any intervention, but because of the response of Ugandans to observed deaths [24].

Data about the effectiveness of STI treatment has also demonstrated mixed results. On the one hand, an early randomized control trial suggested that STI treatment would have a significant impact on the prevalence of HIV [25]. However, subsequent studies suggested that this impact was much less than previously anticipated [26].

Other analyses have produced contradictory results about where current infections are actually occurring. In Zambia, for example, it has been argued that commercial sex is not a significant driver of that country's epidemic, with only 7% of new infections occurring among traditional vulnerable groups (sex workers, clients, truck drivers and uniformed services). The Goals model analysis, however, indicated that commercial sex represents a much more significant problem, with 21% of all new HIV infections within this population [27].

Contradictory results have also been reported in Uganda, where David Wilson of the World Bank has argued that 65% of all new infections occur among married couples, thus justifying an allocation of resources which is more generalized [14]. However, David Apuuli of Uganda's AIDS Commission argues that sex work still represents a major mode of transmission, thus justifying a more targeted approach and a more focused allocation of resources [28]. These results leave policymakers with uncertainty about where new HIV infections will actually occur and how best to allocate limited resources.

#### Multisectoral response

A third possible explanation why resources are not better focused on key interventions is that HIV and AIDS is viewed as a disease requiring a multisectoral response. An example of this argument is contained in the statement below, from the Tanzanian National Strategic Plan (NSP) (2003-2007).

*"All major elements of the National Response have to be in place if the response is likely to achieve its desired impact. The NMSF does not attempt to prioritize among those objectives or strategies. It insists on the comprehensiveness of the response, knowing that due to preferences, experiences, resources available, etc. priorities will have to be established in distinct areas once Operational Plans and Activities are developed. It is one of the most important tasks of TACAIDS as the main guardian of the National Response to ensure that all areas are covered and balances between the areas are maintained or (re-) established" *[29].

This perspective has merit for a number of reasons. It has been shown that a multisectoral response not only generates more resources, but also takes advantage of synergies which can exist when people are approached with HIV and AIDS messages from varying sources (e.g., mass media, peer education, etc.) [30]. An approach which suggests that a country should spend all of its resources on one intervention (e.g., condoms) is unlikely to produce the same benefits as a holistic program which focuses on a wide variety of interventions. Thus, STI treatment, condom distribution, ART, and stigma and discrimination interventions (among other interventions) are likely to have synergistic benefits which could not be achieved if a country merely spent all of its resources on a single, prioritized intervention.

This argument, however, must be balanced against the temptation to scatter resources across all interventions, with little or no priority given to those interventions likely to produce the greatest possible impact. As illustrated by the 1998 UNAIDS Strategic Planning Guidelines, countries should avoid the temptation of using a multisectoral process as an excuse to avoid prioritization.

*"The complexities of HIV sometimes have led governments to attempt planning for all eventualities. Moreover, donors and other external agencies have frequently added their own agendas to already unwieldy plans that cover many areas, resulting in generally low implementation rates, poor performance, and overburdening of scarce national staff. A more strategic approach concentrates on planning in priority areas, through identifying the epidemic's most important determinants" *[31].

#### Political will

Another explanation for why countries don't allocate resources using an evidence-based approach is that countries lack the political will or support to address particular risk groups (for example, see "The Wisdom of Whores", for an extensive explanation of why IDU are seldom reached in Asia, despite their acute vulnerability to HIV) [12]. In many countries in Latin America and the Caribbean, for example, the epidemic pattern suggests that the top-priority population group should be MSM. However, in much of Latin America, there is little support for spending resources on MSM, and thus policymakers may decide to instead focus their resources on addressing the general population.

**Figure 5:**
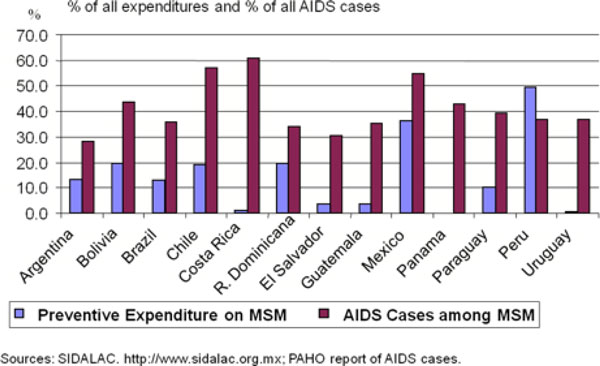
**AIDS Spending on MSM Relative to MSM Cases**.

Figure 5 confirms that MSM represent a large proportion of all AIDS cases in most of Latin America (in Costa Rica, for example, MSM represent more than 60% of all AIDS cases). However, the graphic shows that most prevention resources are not focused on MSM. In fact, in every country except Peru, the analysis suggests that resources are disproportionately small compared to the impact that MSM represent in terms of AIDS cases. In this case, it appears that resources are not being allocated where they can have the greatest possible impact.

The argument about spending resources on the general population rather than already stigmatized groups does have some merit. By focusing attention on MSM, for example, as a primary target population in Latin America, there is a possible risk that MSM would become further stigmatized and blamed for spreading the epidemic. However, a country's national strategic goals are unlikely to be achieved if a predominance of resources is being spent on interventions that focus on relatively low-risk populations, rather than on those subpopulations where the epidemic is actually spreading.

#### Inefficiency

Another possible explanation for why resources are not achieving the expected outputs is that the money is spent on interventions that are delivered inefficiently [9]. Thus, an intervention like voluntary counseling and testing (VCT) may be shown to be highly cost-effective under certain conditions. However, when the resources are actually allocated to those organizations which deliver VCT services, these services are implemented inefficiently, resulting in a less-effective intervention and a poor allocation of resources.

#### Poor planning

In a number of cases, a poor allocation of resources occurs as a result of poor planning. As has already been noted, countries such as Tanzania have avoided explicitly prioritizing interventions in their strategic plan, and have instead passed this responsibility on to their AIDS program [29]. Other countries, such as Nicaragua, identify so many prioritized populations in their strategic plan that the prioritization essentially becomes meaningless (Nicaragua identifies 11 populations in their plan, including young people, children exploited by violence, children exploited by sex, MSM, sex workers, mobile populations, prisoners, uniformed services, pregnant women, indigenous populations, and orphans) [32]. Other NSPs, such as those from Myanmar, prioritize "men and women of reproductive age", which covers such a large proportion of the entire population that it essentially eliminates any prioritization at all [33].

It is also not uncommon to find NSPs which prioritize certain interventions or populations, but then allocates the largest share of funds to interventions which are not prioritized. In Kenya, for example, the Ministry of Health's 1999-2004 HIV/AIDS Strategic Plan indicated that the largest part of the budget should be for the procurement and distribution of STD drugs, even though the plan itself did not emphasize STD drug procurement as a priority in the plan [34].

#### Lack of control over resources

Another possible reason why resources may not be allocated to produce the greatest possible impact is that the country itself cannot control how resources are being spent. For example, some international donors such as PEPFAR have their own agenda to fulfill in regards to allocating resources. In this case, recipient countries are likely to be hesitant to turn down resources, even if those resources will skew the national response towards interventions which planners don't believe will be successful.

In conclusion, there are various reasons why countries may not pursue an "evidence-based allocation strategy". The most common reasons include a lack of data, contradictory results from research, the perceived need for a broad, multisectoral response, various political factors and inefficiency. Each of these reasons has some validity, yet the overall result is that resources are being spent in a way that does not maximize the impact of resources being spent. The next section, therefore, addresses how some countries have used evidence-based tools to better allocate available resources.

## Description of costing and resource-allocation models

As described previously, there are various reasons why resources are not spent more strategically or based on the growing evidence about how best to allocate resources. The following section will review some of the experiences of countries that have tried to improve their resource-allocation strategy.

### Determining resource requirements

While determining resource requirements may be an ongoing process for most countries, the challenge of estimating resource needs becomes most acute when a country is either designing a new (typically 5-year) HIV and AIDS strategic plan or developing a request for resources from donors (most typically the Global Fund).

Unfortunately, the costing of a strategic plan or a Global Fund proposal is usually conducted after all decisions about strategies and priorities have already been made. Thus, the costing usually entails putting a monetary figure on decisions that have already been made, with little or no discussion about how different strategies or priorities may or may not be feasible given the available resources.

The Resource Needs Model (RNM) has been widely used to encourage countries to develop costed plans which are evidence-based [6]. The prevention component of RNM provides a set of global default-unit-cost estimates, which the user is encouraged to modify for their own particular country. RNM also requires users to estimate the size of their target populations and the baseline and planned coverage for their target populations.

Without careful consideration of feasibility, a strategic plan can quickly become a "wish list". An example of this is Zambia's Round 5 Global Fund application. In 2005, Zambia unsuccessfully requested US$1 billion over 5 years from the Global Fund. As it turned out, the 26 countries which did receive immediate approval for their Global Fund applications had requested a cumulative total of $977 million over 5 years. In other words, Zambia had requested funds greater than the combined amount requested by the 26 countries with successful applications. While Zambia is a country that is severely affected by HIV and AIDS, their "wish list" approach to costing its Global Fund proposal concluded with a failure of the country to obtain any HIV and AIDS funds in Round 5.

In the case of South Africa's 2007-2011 NSP, the estimated resource requirements from domestic and international sources were calculated to be R45 billion (approximately US$4.6 billion) [35]. While this seems like a potentially unrealistic target, data from 2007 suggest the country is actually quite close to meeting their first resource-generation target. In 2007, South Africa generated 96% of the NSPs resource requirements (R4.5 billion, up from R4.3 billion in 2006).

However, the resources required are calculated to rise from R4.7 billion in 2007 to R11.3 billion in 2011. South Africa's NSP calls for a 36% increase in spending in 2008 and a 140% increase in spending overall between 2007 and 2011. Thus, South Africa will need to continue to make a substantial increase in its effort to generate new resources and meet the resource generation targets that the plan defines. Is South Africa's NSP a "wish list" or a 'feasible plan"? Only time will tell.

### Allocating scarce resources

When most strategic plans are costed, there is little or no effort to assess what would happen if the levels of resources do not become available. A costed strategic plan should ideally consider alternative scenarios, aligning the priorities to assumptions about various levels of funding actually becoming available. A country, for example, might cost its strategic plan at $2 billion over 5 years, but then quickly realize that the level of funding for the program is unlikely to exceed $1 billion. Should the country cut all of its targets in half? Are there ways to reallocate funds to ensure that the targets can still be achieved despite the limited funds? Which budgetary items are the most critical and therefore should be fully funded, as opposed to those items which are important but not critical?

The Goals model is a key tool in HIV-related strategic planning, evaluation and proposal development. The Goals model is a resource-allocation tool that has been used to answer the following types of questions:

• What resources are required to expand coverage of prevention, care, treatment and mitigation services to all who need them?

• What goals can be achieved with the available resources?

• How can resources be allocated most efficiently to provide the greatest benefit?

• What is the gap between resources required and those available?

• What will be the impact of cuts in current levels of funding?

The Goals model allows users to test alternative patterns of resource allocation and observe how these decisions are likely to affect the overall achievement of specific targets. For example, countries can assess if spending more money on interventions with sex workers is likely to have a greater or lesser impact on the prevalence and incidence of HIV than interventions focused on youth.

**Figure 6:**
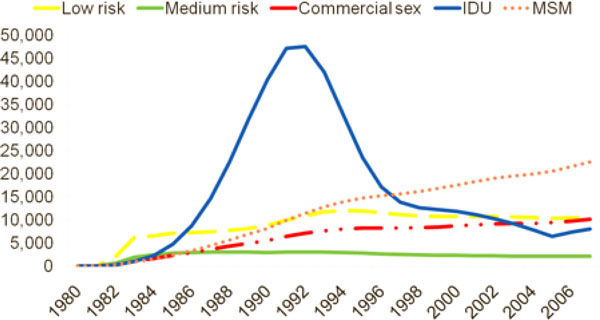
**Source of New HIV Infections in Brazil**.

One challenge with the Goals model, as with all models, is that they can only be effectively used in policymaking with up to date and accurate input data. This is particularly important because it has been observed that the HIV epidemic rapidly evolves. As shown in Figure 6, Brazil initially had an epidemic that was predominantly driven by injecting drug users. However, by 1992, the epidemic among IDU had peaked. Today it is estimated that most new HIV infections are occurring among MSM and sex workers [36].

Thus, if a country is responding to their epidemic using outdated epidemiological information, they are likely to find that their resource-allocation strategy is highly ineffective. Old data from Brazil, for example, would suggest that interventions should be focused on IDU, while more recent data would indicate that more resources should be spent on MSM.

While later sections will focus largely on the experience of countries in using the Goals model, it is worth noting that other resource-allocation models exist and have been applied. These include the ABCE model, an optimization model which focuses on identifying the most cost-effective prevention interventions [37]. ABCE was developed by the World Bank and applied in a number of countries, including India, Honduras and Brazil.

The S4HARA model was developed for the purpose of expanding resource-allocation decisions beyond factors of efficiency, but also tries to take into consideration non-quantifiable factors such as equity [38]. This model has been applied in a South African health clinic, with the result that the authors recommended an increased effort to promote condoms at this site.

## Experience with one resource-allocation tool

The Goals model has been used to improve the resource-allocation process in seventeen countries since 2002, including eight of the fifteen PEPFAR countries. Of the seventeen countries where the Goals model has been used, twelve are in Africa, three are in Asia and two are in Latin America: Uganda, Lesotho, Kenya, South Africa, Ghana, Ethiopia, Namibia, Zambia, Mozambique, Rwanda, Malawi, Mali, Honduras, Mexico, China, Cambodia, Ukraine. All seventeen applications were conducted between 2002 and 2008, with two countries (Ethiopia and Kenya) utilizing the model twice.

The following section provides information on resource-allocation exercises conducted in eight countries between 2002 and 2008 (Uganda, Lesotho, Kenya, South Africa, Honduras, Namibia, Zambia, Ethiopia and the Ukraine). These eight country examples were selected and discussed in this report because of their geographic diversity, as well as the illustrative "lessons learned" from these examples. The purpose of this review is to focus on the key experiences from countries which have tried to better allocate their HIV and AIDS resources.

### Lesotho

In 2000, the government of Lesotho completed its 2001-2003 NSP, which was estimated to cost a total of US$1 billion, or US$333 million per year [39]. While Lesotho has an extremely high prevalence of HIV (currently estimated to be 23%), the size of the population is relatively small (approximately 2 million). Thus, many of the international donors viewed the costed strategic plan to be highly unrealistic, particularly given that the levels of resources available in Lesotho were only US$10 million per year at the time. The Goals model exercise in Lesotho was conducted in 2002, in large part because the NSP was not successfully generating the required resources.

By using the Goals model, the costing of the NSP was revised [40]. Working with a team of local costing experts, it was possible to revise the budget downwards from US$333 million annually to US$100 million annually. This was achieved largely by reevaluating unrealistically large cost estimates for two interventions: STI treatment and the distribution of pamphlets.

A new priority setting team was then developed and assigned responsibility for ensuring that the budget was consistent with the NSP. The team identified interventions that could achieve their goals at a much lower cost. For example, the priority setting team noted that the budget for community mobilization was excessive and they were able to propose changes which would make this intervention much less costly. By identifying those items which were most critical for Lesotho's national response, it was possible to develop a new budget of approximately US$40 million per year, or 12% of the original costing.

This new costed plan may have appeared to be less ambitious, but in reality it still represented a 4-fold increase in spending. As a result, Lesotho was able to use this information to successfully apply for additional donor funding. In 2003 Lesotho requested and received US$29 million in additional HIV and AIDS funds from the Global Fund. In 2006, Lesotho received approval for an additional $40 million of HIV and AIDS funds and in 2008 Lesotho successfully requested another US$33 million.

The key lesson learned from this resource-allocation exercise appears to be that developing more realistic, yet still ambitious, budgets is likely to produce more funds than developing a "wish list" approach to resource allocation. The Goals model was successful in getting policymakers from Lesotho to recognize that HIV and AIDS spending remains limited, and therefore a more prioritized resource-allocation strategy was required. As a result, Lesotho was successful both in prioritizing and generating new resources.

### Kenya

The Goals model was used in Kenya both in 2002 and in 2004 [41,42]. The 2002 application was conducted as part of the country's Joint AIDS Program Review. The 2002 application provided the country with an analysis with recommendations both for generating new resources and reallocating current resources. Kenya's NSP (2000-2005) indicated that the country hoped to reduce HIV prevalence by 20% to 30% among those 15 to 24 years old [43]. The country estimated that existing commitments from donors and the Kenyan government amounted to approximately US$710 million over 5 years.

The modeling exercise revealed that Kenya's HIV and AIDS response was succeeding at reducing HIV prevalence, but that the reductions were probably not going to reach the expected 20%-30% target by 2005. The modeling exercise did reveal that the target was still feasible if the country was able to both reallocate existing resources and obtain new funds. The allocation exercise indicated that interventions with sex workers, STI treatment, workplace interventions and voluntary counseling and testing were particularly cost-effective. Conversely, there was much less evidence that interventions such as community mobilization, outreach to out-of-school youth and mass media would have much of an impact in reaching Kenya's targets.

In terms of the country's treatment goals, the modeling exercise related the country's treatment target for 2005 (60,000 people on ART) to the resources required (estimated to be between US$64 and US$76 million). By calculating more carefully the resource requirements, Kenyan policymakers were better prepared to assess their resource needs and to successfully seek out these resources.

The result of this exercise was that the country was able to submit a successful Global Fund application which defined why additional resources were required both for prevention and treatment. Using data from the Goals model, the application was also able to more accurately define what could be achieved with the resources requested. A total of $107 million was approved in 2003 for Kenya from the Global Fund.

The resource-allocation exercise was less successful at getting the country to reallocate its HIV and AIDS funds. In many ways, the country viewed the exercise as important for obtaining additional funds, but less important in terms of developing priorities and ensuring that those priorities were actually achieved. Furthermore, as new PEPFAR funds became available, Kenya appeared to be less concerned about issues of sustainability.

In 2004, the National AIDS Control Council began preparation of its new strategic plan for 2005-2010. A team working on resource allocation applied the Goals model to estimate the resources that would be required to fully scale up prevention and treatment programs, and the impact that could be expected if full scale-up were achieved. Significant effort was put into collecting up-to-date unit costs for prevention and treatment programs implemented by the Government of Kenya and by other implementing organizations. The resulting goals formed part of the vision statement for the plan. The resources required exceeded the expected availability of funds and formed the basis for a renewed effort at resource mobilization.

### South Africa

The Goals model application in South Africa was conducted in 2003, half-way through the country's 2000-2005 NSP [44]. The modeling exercise was pursued to assess if the country needed to make any modifications in the way resources were being spent [45].

The analysis did find a number of areas where additional funds should be spent, including interventions with sex workers, MSM and IDU. The analysis also showed that funding for condoms was insufficient, and that the government should allocate additional resources to distribute additional condoms. The Goals model was also used to estimate the cost of a national PMTCT program; confirming previous cost estimates for this intervention. Finally, the Goals model confirmed that the general provision of ART would be affordable for the government.

There were numerous positive outcomes based on these observations. For example, the government prioritized and allocated additional funds for sex-worker interventions, condom distribution, PMTCT programs and general ARV access. However, while the government agreed to research the needs of MSM and IDU in South Africa, they did not agree to additional funding to reach these populations. In other words, despite some evidence to the contrary, South Africa remained unwilling to address existing political barriers which limited their ability to work with sensitive subpopulations such as MSM.

### Honduras

In the 2004 Goals model application, an assessment of the country's NSP (2003-2007) was performed; the model was used to assess the impact of a potential decision by the Global Fund to discontinue funding the Honduran HIV and AIDS program [46]. This application was conducted because there were serious concerns that Global Fund would discontinue funding in Honduras for its HIV and AIDS program.

The NSP indicated that Honduras required US$25 million over the 5-year life of the plan [47]. There was general consensus that the resources budgeted in the country's NSP were insufficient to achieve the goals established in the plan. Therefore, various scenarios were evaluated for developing a more ambitious plan for addressing HIV and AIDS in Honduras.

An analysis of the resources required to fully cover all HIV and AIDS services and reach all vulnerable subpopulations indicated that US$285 million would be required over the 5 years of the plan. This full-coverage scenario was deemed unlikely to be feasible.

A third scenario was developed which would require that the Global Fund continue their funding of the Honduras program. In this scenario, Honduras would have access to US$58 million over 5 years. This scenario was estimated to result in a 14% reduction in HIV prevalence and a 51% reduction in HIV incidence. The expected number of HIV infections averted was 22,000, at a cost of US$1,100 per infection averted.

The analysis was effective at demonstrating to the Global Fund that their contribution to the HIV and AIDS response was critical and was likely to have significant benefits, especially in terms of infections averted. As a result of the analysis, along with various commitments from the government of Honduras, the Global Fund decided to continue funding of the Honduran HIV and AIDS program.

### Namibia

A Goals modeling exercise was conducted in Namibia, a year after the start of their NSP (2004-2009) [48,49]. When Namibia launched its NSP, it lacked information about its current HIV and AIDS expenditures, although the country was able to develop an estimate of its resource needs. By 2005, the country was able to calculate its resource available at US$79 million, an amount which was expected to rise to $130 million by 2007. The challenge for Namibia was that the NSP estimated that the resources required in 2007 were only $124 million. In other words, the data appeared to suggest that Namibia had access to higher levels of resources than were actually needed in 2007.

The exercise in Namibia was designed to: 1) assess if the NSP had accurately been costed and 2) to evaluate how additional resources might be allocated to achieve the greatest possible impact.

The modeling indicated that a more realistic costing of the country's NSP would require approximately $175 million to reach the desired coverage of services. It was determined that, relative to a scenario of constant funding from 2005 to 2009, HIV incidence would be reduced from 2.26% to 1.63%. The additional funding would result in more than 16,000 HIV infections averted. The proposed funding would further decrease HIV prevalence by a small amount (from 19.3% to 18.6%). The model calculated that ART coverage could be increased from 21% to 58%.

The exercise in Namibia confirmed what the government already suspected, which was that the NSP had been significantly undercosted. While the government of Namibia recognized that the Goals model could help to improve their costing, they did not choose to revise the costing of the NSP.

Namibia is currently beginning to develop a new NSP, and have indicated a desire to conduct a new Goals model application which will improve the country's ability to devise more tenable cost estimates. Policymakers also hope that more realistic cost estimates will assist the country in generating new resources for its NSP and achieve universal access for prevention and treatment services.

### Zambia

In 2005, Zambia requested support in learning about and utilizing the Goals model [27]. As previously indicated, Zambia had earlier in the year submitted an unrealistically ambitious and unprioritized Global Fund application. However, at the time of the Goals application in 2005, Zambian policymakers could point to a number of important successes, including reductions in HIV prevalence, increases in condom use and growing access to free ART throughout the country. Furthermore, Zambia benefitted from a significant growth in the availability of resources from PEPFAR, the Global Fund and the World Bank.

Zambia at the time of the Goals application was completing its HIV and AIDS strategic plan for 2002 to 2005 [50]. It was also in the process of designing its new strategic plan for 2006 to 2010 [51].

Three scenarios were developed for the Zambian government. In the first, universal access would be achieved by 2009 for ARVs, PMTCT and blood screening, but there would be no additional spending on prevention programs. This first scenario would require that Zambia increase its annual HIV and AIDS expenditures from US$100 million per year in 2004 to US$260 million per year by 2009. Of this amount, the largest expenditures, by far, would be on the provision of ART (estimated to cost US$190 million by 2009). This treatment-intensive scenario would result in HIV prevalence rising, from the projected prevalence of 13.3% in 2009 to 15.4%. HIV prevalence rises as ART scale-up occurs due to the increased life expectancy of those on treatment. However, this rise in prevalence can in part be counteracted with a corresponding increase in prevention scale-up.

The second scenario assumed universal access would be achieved for all prevention, care, treatment and mitigation interventions by 2009. This full-coverage scenario resulted in only a slight increase in HIV prevalence (13.7% in 2009). However, this second scenario was estimated to cost much more than the first scenario (US$442 million in 2009).

The third scenario assumed that the targets for the first scenario would be achieved, but an additional US$30 million would be made available for Orphans and Vulnerable Children (OVC) interventions and the most cost-effective HIV prevention interventions. This third scenario was projected to result in Zambia spending a total of US$290 million in 2009, with prevalence rising to 14.2%.

A review of these three scenarios indicates a number of important observations regarding the allocation of HIV/AIDS resources in Zambia. Scenario 1 provides some indication regarding the cost of providing full coverage for the 3 identified services. While this scenario was likely to represent a significant achievement, it also suggests a concern because of rising levels of HIV prevalence. While Scenario 2 was likely to produce the best possible results, it remained unclear if this level of increased funding was likely to become available. Finally, Scenario 3 suggested that an allocation of resources which balances prevention, care and treatment - and invests in those interventions that are likely to have the largest impact - will probably produce extremely impressive results, without requiring the very high costs required of Scenario 2.

While Zambia did not adopt all the conclusions from the Goals report, Zambia's national strategic plan did indicate that US$255 million would be required in 2009, an amount very similar to that calculated in Scenario 1. Furthermore, the strategic plan noted that the "Availability of ART will decrease the mortality rate and, therefore, in the short to medium term prevalence could in fact increase as ART becomes more readily available." A similar conclusion was made from the Goals model exercise, with all three scenarios resulting in a projected increase in the prevalence of HIV.

### Ethiopia

The Goals model in Ethiopia was first applied in 2006, sixteen months after the country had launched its 2004-2008 NSP [52,53]. The Goals model is currently being updated for use with the upcoming 2009-2013 National Strategic Plan. While specific data on HIV and AIDS spending in Ethiopia are not available, it was estimated that Ethiopia spent approximately US$110 million on HIV and AIDS interventions in 2005. This level of funding represents a substantial effort on the part of the Ethiopian government and international donors. However, this funding was only enough to achieve relatively low levels of coverage for most HIV and AIDS services.

The country's 2004-2008 NSP indicated that approximately US$170 million per year is required to achieve the desired targets. This would represent an increase of more than 50% from the estimated 2005 level of resources. However, the Goals model calculations suggest that this increase in funding would still not be sufficient to achieve universal access.

The Goals model exercise in Ethiopia indicated that universal access (defined as 80% coverage for all services) could be achieved in 2010 with an 8-fold increase in spending, reaching US$1 billion per year by 2010. However, a review of the country's capacity constraints, as well as access to donor resources, suggested that this type of rapid growth was unlikely to be achieved in such a short period of time.

Another scenario reviewed involved prioritizing interventions in the costed strategic plan (to achieve greater coverage in certain areas) and also striving for higher levels of funds. In this scenario, Ethiopia would need to generate US$300 million in 2010, triple the level of 2005 funding but less than a third of the resources required for full coverage. The result would be to decrease prevalence (from 5.3% to 5.1%) and incidence (0.78% to 0.60%) and provide ART coverage to 230,000 people infected with HIV. Of all the scenarios, this seemed to be the one which created the best combination of feasibility and ambition.

It should be noted that Ethiopia's first Goals model application utilized the epidemiological data and prevalence trends that were available in March 2006. The Goals model concluded that, without additional behavior change, prevalence would rise from 4.7% in 2005 to 6.5% in 2010. However, when the 2005 Demographic and Health Survey (DHS) and Antenatal Surveillance (ANC) data became available later in 2006, it revealed that prevalence among 15-49 year olds was between 1.4% (DHS) and 3.5% (ANC), both of which were well below the estimates used in the Goals report. Following an extensive analysis of the DHS and the ANC data, Ethiopia agreed on a single point estimate of 2.1%, less than half of the prevalence calculated when the first Goals application was performed. Thus, in 2009, the calculations are being revised and will be used as an input into Ethiopia's next NSP.

There are a number of important lessons learned from this exercise. First, it appears that the 2004-2008 NSP was not ambitious enough, despite a projected 50% increase in the calculation of resources needed. On the other hand, the resources needed to ensure universal access of services in Ethiopia ($1 billion in 2010) were determined to be infeasible, given capacity constraints and an unrealistically rapid growth in donor funding. Thus, by conducting a Goals modeling exercise, it was possible to develop a scenario that would achieve ambitious levels of coverage while still targeting resource levels that were likely to be achievable.

Another lesson learned from the Ethiopia exercise involved the importance of having accurate epidemiological data. As noted above, the Goals model was conducted at a time when it was determined, based on ANC data, that HIV prevalence was nearly 5%. Subsequent data obtained from the country's 2005 DHS and ANC data suggest that prevalence was actually closer to 2%. This will clearly have implications on the resource estimates for achieving universal (or near universal) access.

### Uganda

In 2006, the Uganda AIDS Commission (UAC) began to prepare its new HIV/AIDS Strategic Plan for the years 2007/8 - 2011/12 [54]. The work began with a review of the accomplishments and failures of the previous plan, collection of up-to-date data and a situation analysis. The costing and resource-allocation work started early in the process with an assessment of current spending and estimates of future resource requirements. The assessment of current spending found that resources available for HIV and AIDS programs had increased from $39 million in 2003/4 to $170 million in 2006/7. The team working on resource allocation held discussions with donors and the UAC to estimate the likely future trend in resource availability. The team developed several future funding scenarios based on a detailed analysis of donor plans and government budgets. Based on increased funding from PEPFAR, Global Fund and domestic sources, resources available were projected to increase to $350-$500 million per year by the end of the plan.

The team used the Goals model to estimate the future funding required to scale up coverage of all key prevention, treatment and mitigation interventions to reach full coverage by the end of the plan [55]. They estimated that about $620 million would be needed in 2011/12. Thus, the gap between resources needed and resources expected to be available was $120-$270 million in 2011/12. Since it was clear that funds would not be available to scale up all interventions, decisions needed to be made on what was most important to fully fund and what would have to be only partially funded.

The resource-allocation team used the Goals model to estimate the impact of different allocation scenarios. Impact was measured by several indicators, including two for prevention (the number of new infections, the reduction in annual incidence), two for treatment (ART coverage and the number of AIDS deaths) and one for mitigation (coverage of support services for orphans and vulnerable children). Several different allocation schemes were analyzed, including:

• Prevention first: fully fund all prevention programs, allocate the remaining funds to treatment and mitigation

• Cost-effective prevention first: fully fund the most cost-effective prevention interventions, keep other prevention constant, allocate the remaining funds to treatment and mitigation

• Treatment first: achieve universal access to treatment, allocate the remaining funds to prevention and mitigation

• Mitigation first: increase funding for OVC programs by 6-fold, allocate the remaining funds to prevention and treatment.

Allocation scenarios that prioritized prevention prevented many new infections but did not have as much impact on reducing deaths or mitigating the effects on children. Allocating funding to treatment averted the most deaths and achieved high ART coverage but did not prevent as many new infections.

This information was presented in meetings with the UAC, civil society, donors, government departments, and parliamentary committees. At these meetings, participants discussed the benefits of each allocation strategy and voiced their opinions about priorities for the new plan. This had happened before but what was different this time was that these discussions were informed by information on the impact of their decisions on key indicators of impact. After much discussion, it was decided to give top priority to fully funding the most cost-effective prevention strategies.

### Ukraine

Ukraine has the most severe epidemic in all of Europe, with a prevalence estimated to be 1.63% [56]. Estimates indicate that a third of male mortality and more than half of female mortality among those between the ages of 15 and 49 in Ukraine will be attributable to AIDS within the next five years.

Ukraine is preparing for their next HIV and AIDS strategic plan, which will run from 2009 to 2013. As part of that preparation, a team applied the Goals model to evaluate a number of different scenarios. These scenarios include:

1. Constant expenditures: Funding remains constant from 2008 to 2013.

2. Universal access: Funding increases to achieve universal access.

3. Prevention focus: Universal access is achieved for prevention; treatment spending increases by 50%.

4. Treatment focus: Universal access is achieved for treatment; prevention spending increases 50%.

5. Limited funding: Budget based on the commitment of the Ministry of Finance and the Global Fund.

Each of these five scenarios was evaluated in terms of cost, impact on incidence and prevalence, and levels of coverage for care and treatment services. The results are shown in Table 1. Scenario 1 is shown to be the least expensive, but also achieves the lowest level of coverage for ART and for prevention coverage (IDU drug substitution coverage is used as an indicator of prevention success). Scenario 2 is likely to have the greatest impact in terms of both ART and prevention coverage, but this scenario is the most expensive. Scenario 3 is likely to have the greatest impact on HIV prevalence, but it only achieves 44% coverage for ART. Scenario 4 achieves high level of treatment coverage, but results in the highest prevalence of HIV. Finally, scenario 5 also reaches high levels of treatment coverage, but only achieves 38% of IDU with drug substitution programs.

**Table 1:** Scenarios for Resource Allocation

#	Scenario	Cost in 2013	**ART Cov**.	**IDU Cov**.	Prevalence
1	Constant funding	$118 million	16%	0%	2.24%
2	Universal access	$307 million	80%	60%	2.26%
3	Prevention focus	$242 million	44%	60%	2.18%
4	Treatment focus	$270 million	80%	30%	2.32%
5	Limited funding	$244 million	80%	38%	2.26%

The result of the exercise was to provide Ukrainian policymakers with different scenarios which relate resources to the achievement of different targets. While the Ministry of Finance had originally proposed that the country pursue Scenario 5, there was a recognition following the exercise that the level of funding was insufficient to achieve the country's goals. As a result, the Ukrainian Ministry of Finance agreed to higher levels of government funding. In addition, the Ukrainian Ministry of Health agreed to conduct further negotiations with ARV manufacturers, with the intent of lowering the price of ARVs in the country.

## The future of resource allocation

This final section discusses the possible future of resource allocation in the field of HIV and AIDS, including improvements in existing resource-allocation tools, the improved willingness of countries to link priorities and resources, the barriers that lie ahead for improving the resource-allocation process, and some key actions that could be pursued in the next few years to overcome those barriers.

### How can resource allocation be made better?

There are various ways in which countries can be encouraged to think beyond their current paradigm regarding HIV and AIDS resource allocation. Perhaps the first step in allocating resources effectively is to ensure that policymakers truly understand their own epidemic. In other words, it is critical to assess not only where HIV infections have occurred (in terms of vulnerable subpopulations, regional variations, etc.), but also to understand where the next infections are likely to occur. Countries need to move beyond the oversimplified conclusion that "everyone is at risk", and instead truly understand whether certain subpopulations are at greater risk than others. UNAIDS has been supporting a series of regional activities known as "Know Your Epidemic, Know Your Response" in which country teams assemble information on the status of the epidemic and their national program. The Modes of Transmission model is applied to estimate the sources of new infection. The results from this modeling are compared to the current allocation of efforts to determine how the response might be improved.

Next, countries need to have a clear grasp on the costs of different interventions. In the earlier years of the epidemic, it may have been acceptable that countries lacked information about a reasonable range of unit costs for a set of prevention and treatment interventions. However, at this stage of the epidemic, it is totally unacceptable that countries cannot provide even a reasonable range of unit costs for particular interventions. Where unit cost data are not available, it should be a high priority to collect such information. Most organizations implementing interventions know their own costs, since they need to prepare budgets to request support from funding agencies. This information needs to be compiled systematically and used to estimate resource requirements. This is important not only for the purpose of allocating resources, but also for identifying potential inefficiencies in scaled-up programs.

A key way to improve their resource-allocation process is to integrate resource allocation into each country's entire planning process. Most countries assess their resource needs as a final step in the planning process, rather than throughout the overall planning process. This is not to suggest that the planning process should be limited by some arbitrary financial limits. However, countries should be encouraged to consider throughout the planning process a set of different scenarios based on assumptions about what level of resources may be realistically available.

Finally, countries should design their own plan for financial sustainability. This is important because most countries today plan no further than their next budget cycle, paying very little attention to how the priorities of donors and governments might shift in the future. This is particularly critical for countries which rely heavily on a small number of donors (e.g., PEPFAR; Global Fund, etc.). All plans should include contingencies to cover worst-case scenarios, which is particularly important given the current global financial crisis. What happens if PEPFAR funding is reduced or "flat-lined"? What happens if a country doesn't win any further Global Fund applications? What if the priorities of a country shift away from HIV and AIDS?

### Incorporating new information into resource-allocation models

The future of evidence-based planning will ultimately require that existing tools that are used to address issues of resource allocation are greatly improved and that new tools are developed. A critical first step in improving resource-allocation tools involves improving knowledge about the costs and cost-effectiveness of interventions. Existing tools rely on an incomplete database of cost and cost-effectiveness studies. Little is known, for example, about the cost-effectiveness of community outreach interventions or interventions designed to reach MSM. A concerted global effort should be made to expand the current literature on the cost and cost-effectiveness of HIV and AIDS interventions, with an emphasis on providing data that can specifically be used by policymakers as they allocate resources.

It is also imperative that resource-allocation tools reflect current knowledge about new interventions. For example, the Goals model has recently been updated to reflect new data regarding the effectiveness of male circumcision. As future interventions become available (e.g., microbicides, vaccines, etc.), resource-allocation tools need to remain current with these new interventions.

Next, the way in which treatment and prevention are linked remains poorly understood. For example, little is known about the extent to which increased access to treatment will affect the future incidence of HIV. It is inevitable that as access to ART increases, the prevalence of HIV will similarly rise. However, it is much less clear how this will affect new HIV infections [57,58]. Further analysis should be performed in developing countries that have had long-term experience offering ART (e.g., Costa Rica, Brazil) to assess how this may already be affecting HIV prevalence and incidence.

Resource-allocation models also need to better take into consideration synergies between interventions. Mathematically, synergies are difficult to adequately model, but the accuracy of future projections depends critically upon the way that these models work. Experience shows that spending large sums of money on only one intervention is unlikely to be successful. The success of STI treatment, for example, depends critically on the effectiveness of condom distribution programs. The uptake of VCT is likely to be strongly affected by efforts to limit stigma and discrimination, and by treatment availability [59]. Increasing government spending on vulnerable and highly stigmatized groups (e.g., MSM) is only likely to occur if resources are also spent on convincing political leaders about the importance of reaching these subpopulations. Each of these types of synergies needs to be considered in the design of future resource-allocation tools.

There also remain areas where significant additional research needs to be conducted. For example, little is known about the resource-allocation strategies of countries which have been able to successfully address the HIV and AIDS epidemic. Up until recently, the lack of such research was understandable, given the paucity of data about the way resources are spent and the very limited information about the historical prevalence trends in various countries. However, at this point in time, there is an increasing level of data about both spending and prevalence trends. This information should be rigorously analyzed so that countries are better able to make resource-allocation decisions.

## Conclusion

This paper was designed to challenge national policymakers to consider how resource allocation is being conducted, and to reevaluate how it might be pursued better in the future. As seen from numerous countries, there are few outstanding examples of countries which have carefully assessed their resources allocation strategy and acted in a way which could be considered "evidence-based". Furthermore, few countries have incorporated into their own strategic planning process an assessment of financial sustainability. Given the current global financial crisis, countries cannot afford to ignore the issues of accountability and sustainability [8].

How can these problems be addressed? A review of current efforts to understand and improve the resource-allocation process indicates both successes and failures. Countries have seen resource-allocation modeling as merely another tool imposed upon them by international donors, rather than an essential process that should be integrated into the country's planning process. Few if any countries have taken the opportunity to conduct resource-allocation modeling so that they can actually shift resources from low priority interventions to those which are a higher priority.

However, countries such as Lesotho, Kenya and Honduras have used resource-allocation modeling as a way to demonstrate to donors the potential benefits of investing in the country's HIV and AIDS response. Countries such as South Africa and Ukraine have used resource-allocation modeling as a way to increase domestic commitment and to generate new resources. Namibia has used resource-allocation modeling to confirm that their NSP was severely undercosted. Uganda used modeling to provide the information for a vigorous debate among stakeholders about priorities for allocating scarce resources.

For the national resource-allocation process to be improved over the long term, both countries and the global community must move forward. Research must be performed on costing and cost-effectiveness. Much more is needed in terms of understanding how resource allocation has helped countries to develop an effective response, while also understanding how poor resource-allocation decisions have limited the impact of available resources.

## Competing interests

The authors declare that they have no competing interests.

## Authors' contributions

SF was responsible for the overall writing of this paper with review and additional input provided by Lori Bollinger and JS. The description of the resource allocation models was written largely by LB and JS. The country-specific Goals applications were conducted by a combination of the three authors.

## List of abbreviations used

ABCE: (Allocating by Cost-Effectiveness Model); AIDS: (Acquired Immune Deficiency Syndrome); ANC: (Antenatal Clinic Survey); BCC: (Behavior Change Communication); DHS: (Demographic and Health Survey); ART: (Antiretroviral Therapy); HIV: (Human Immunodeficiency Virus); IDU: (Injecting Drug Users); PMTCT: (Prevention of Mother to Child Transmission); MAP: (Multi-Country HIV/AIDS Program); MARPs (Most At-Risk Populations); MSM: (Men Who Have Sex with Men); NASA: (National AIDS Spending Assessment); NMSF: (Tanzanian National Multi-Sectoral Framework); NSP: (National Strategic Plan); OVC: (Orphans and Vulnerable Children); PEPFAR: (President's Emergency Plan for AIDS Relief); PMTCT: (Prevention of Mother to Child Transmission); RNM: (Resource Needs Model); STI: (Sexually Transmitted Infection); TACAIDS: (Tanzania Commission for AIDS); UNAIDS: (United Nations Joint Programme on HIV/AIDS); UNGASS: (UN General Assembly Special Session on HIV: and AIDS); VCT: (Voluntary Counseling and Testing).
